# Increased expression of the small lysosomal gene *SVIP* in the *Drosophila* gut suppresses pathophysiological features associated with a high-fat diet

**DOI:** 10.1242/bio.061601

**Published:** 2025-01-30

**Authors:** Brennan M. Mercola, Tatiana V. Villalobos, Jocelyn E. Wood, Ankita Basu, Alyssa E. Johnson

**Affiliations:** Louisiana State University, Department of Biological Sciences, Baton Rouge, LA 70803, USA

**Keywords:** SVIP, Lysosomes, Autophagy, Obesity, *Drosophila*, High-fat diet

## Abstract

Lysosomes are digestive organelles that are crucial for nutrient sensing and metabolism. Lysosome impairment is linked to a broad spectrum of metabolic disorders, underscoring their importance to human health. Thus, lysosomes are an attractive target for metabolic disease therapies. In previous work, we discovered a novel class of tubular lysosomes that are morphologically and functionally distinct from traditionally described vesicular lysosomes. Tubular lysosomes are present in multiple tissues, are broadly conserved from invertebrates to mammals, are more proficient at degrading autophagic cargo than vesicular lysosomes, and delay signs of tissue aging when induced ectopically. Thus, triggering tubular lysosome formation presents one mechanism to increase lysosome activity and, notably, overproduction of the small lysosomal protein, SVIP, is a robust genetic strategy for triggering lysosomal tubulation on demand. In this study, we examine whether *SVIP* overexpression in the fly gut can suppress pathophysiological phenotypes associated with an obesogenic high-fat diet. Indeed, our results indicate that increasing *SVIP* expression in the fly gut reduces lipid accumulation, suppresses body mass increase, and improves survival in flies fed a high-fat diet. Collectively, these data hint that increasing lysosomal activity through induction of tubular lysosomal networks, could be one strategy to combat obesity-related pathologies.

## Teaser

**Summary:** Increasing lysosome activity in the fly midgut offsets negative effects of an obesogenic high-fat diet.

## INTRODUCTION

Obesity has become a major global epidemic; it is estimated that two out of every five adults are overweight or obese worldwide (NCD Risk Factor Collaboration (NCD-RisC), 2017). Long-term obesity is linked to numerous metabolic health conditions, including metabolic syndrome, type 2 diabetes, cardiovascular disease, and cancer (Zhang et al., 2014). However, the genetic landscape of individuals can have a major impact on metabolic resiliency and the ability to cope with metabolic stress (Reed et al., 2010; O'Rahilly and Farooqi, 2006); in fact, some individuals that are overweight or obese never exhibit metabolic disorders, suggesting that they may have genetic factors that offer a protective advantage (Eckel et al., 2016; Stefan et al., 2013; Meigs et al., 2006). Thus, it is conceivable that certain genetic factors could be tapped to improve obesity tolerance in the general population.

One attractive cellular target for improving metabolic resiliency under extreme diet conditions is the lysosome. Lysosomes are major cellular depots that degrade toxic waste products and are an integral part of nutrient sensing and metabolism (Ballabio and Bonifacino, 2020; Settembre et al., 2013). Indeed, lysosome dysfunction has been linked to a wide range of metabolic diseases, including lysosomal storage diseases, diabetes mellitus, and autoimmune diseases (Platt et al., 2012; Malicdan et al., 2008; Gros and Muller, 2023). While the mechanistic link between lysosome signaling and dietary restriction has been studied extensively in recent years (Settembre et al., 2013, 2011; Sancak et al., 2010), how lysosomes respond to nutritional overabundances, such as high-fat and high-sugar diets, remains unknown. Moreover, whether boosting lysosome function can combat obesity-related pathologies has not been thoroughly explored.

In previous work, we found that certain tissues naturally possess lysosomes that are more proficient at degrading complex cargo (Bohnert and Johnson, 2022). For example, in *Drosophila*, muscle lysosomes exist in a constitutive tubular network that degrades autophagic cargo more robustly than other tissues that do not possess natural tubular lysosomal networks (Johnson et al., 2021, 2015). In the *Caenorhabditis elegans* gut, lysosomes are vesicular under basal conditions, but transform into tubular networks upon certain metabolic stimuli, such as dietary restriction (Villalobos et al., 2023; Dolese et al., 2022; Ramos et al., 2022). Notably, lysosomes that form tubular networks are more proficient at degrading autophagic cargo compared to their vesicular counterparts (Villalobos et al., 2023). Thus, altering lysosome morphology represents one mechanism by which lysosomes can increase their activity and digestive capacity.

In ongoing genetic screens, we have discovered key genes that are essential for triggering tubular lysosome formation and demonstrated that overexpressing some of these key genes in other tissues that do not naturally possess tubular lysosomes can stimulate lysosome tubulation ectopically. Of these key genes, the small adapter protein SVIP seems to be the most penetrant and versatile molecule to increase lysosomal tubulation (Johnson et al., 2021; Villalobos et al., 2023). For example, although *C. elegans* do not possess a known *SVIP* ortholog, expression of *Drosophila* or human *SVIP* in the *C. elegans* gut induces tubular lysosomes constitutively, increases autophagic turnover, and improves organismal health (Bohnert and Johnson, 2022; Gill et al., 2024; Villalobos et al., 2023). Thus, *SVIP* could be harnessed to increase lysosome activity in a variety of biological contexts and may be a viable target to treat metabolic diseases.

In this study, we examine how lysosomes in the *Drosophila* gut naturally respond to high-fat and high-sugar diets, and whether increasing lysosome activity can suppress negative physiological phenotypes associated with obesogenic diets. We find that a high-fat diet (HFD) induces enlarged lysosomes in the gut and this correlates with increased lipid content in the gut. Remarkably, we find that increasing lysosome activity via *SVIP* overexpression suppresses fat accumulation in the gut, prevents an increase in body mass, and increases survivability of flies fed a HFD. Collectively, these data suggest that strategies targeting lysosome activity, specifically in the gut, may be a viable means for treating metabolic disorders.

## RESULTS

### A high-fat diet induces an increase in lysosomal area in the fly midgut

**Fig. 1.**
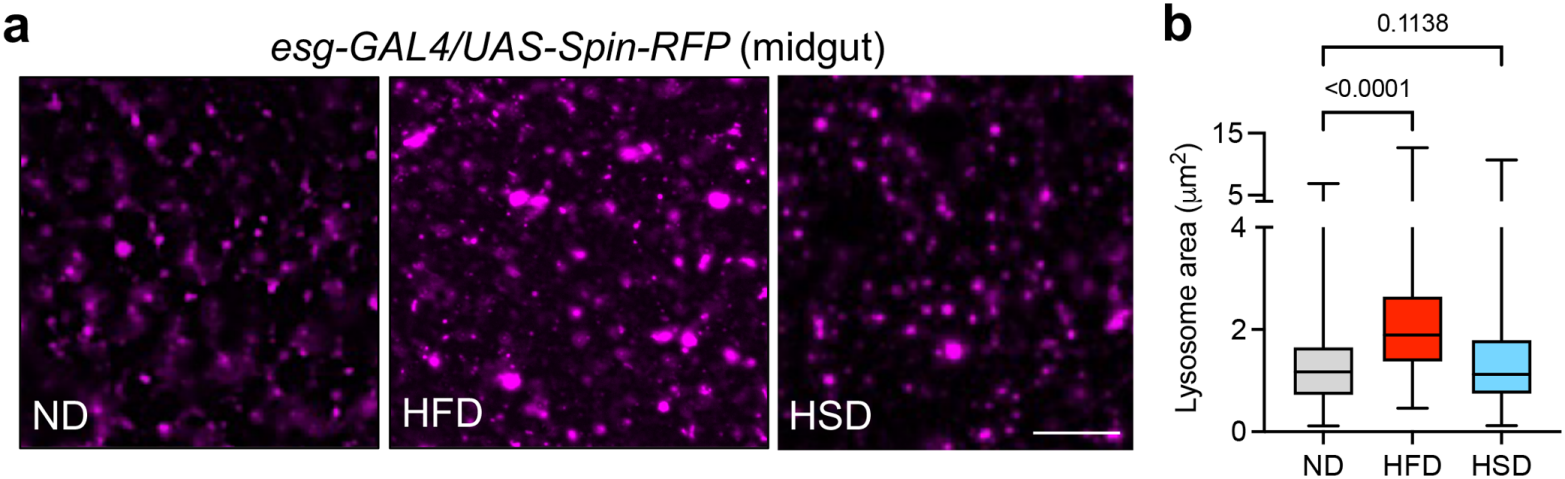
**A high fat diet induces an increase in lysosomal area in the fly midgut.** (A) Representative images of Spin-RFP in the midgut of flies fed a normal diet (ND) or high fat diet (HFD). Scale bar: 5 µm. (B) Quantification of lysosomal area in the midgut of flies fed a normal diet or high fat diet (*n*=200 lysosomes from 10 independent animals for each group). Data are presented as box-and-whisker plots, with horizontal lines inside boxes indicating medians, box edges representing 25th and 75th percentiles, and whiskers extending to minima and maxima. Statistical significance was determined using an unpaired *t*-test (*P*-value on graph).

The fly midgut is the major site of dietary absorption and metabolism of macronutrients into metabolic intermediates that are excreted into the hemolymph (analogous to the mammalian circulatory system) for use by other tissues and organs (Miguel-Aliaga et al., 2018). Consequently, we might expect lysosomes in the midgut to be more highly active in this tissue and have the most effect on fat deposition to other parts of the body. Thus, we first examined potential effects of high-fat and high-sugar diets on lysosome morphology in the fly midgut. Flies were fed a high-fat or high-sugar diet consisting of 15% coconut oil or 30% sucrose, respectively, for 2 days before gut tissues were dissected from live animals and imaged. These diets have been previously characterized to induce obesity and the associated pathophysiological symptoms in *Drosophila* (Yan et al., 2023; Galikova and Klepsatel, 2018; Birse et al., 2010; Musselman et al., 2011). Lysosomes were visualized in live gut tissues by driving expression of the lysosomal membrane protein Spin-RFP (Sweeney and Davis, 2002) in the gut using the *escargot* promoter (*esg-GAL4*). Notably, we observed a significant increase in lysosomal area in flies that were fed a HFD (Fig. 1A,B). In contrast, we did not observe a significant change in lysosome area in the gut of flies fed a high sugar diet (HSD) (Fig. 1A,B). Therefore, we focused our subsequent analyses on HFD only.

### *SVIP* overexpression in the gut suppresses HFD-induced lipid droplet size increase

**Fig. 2.**
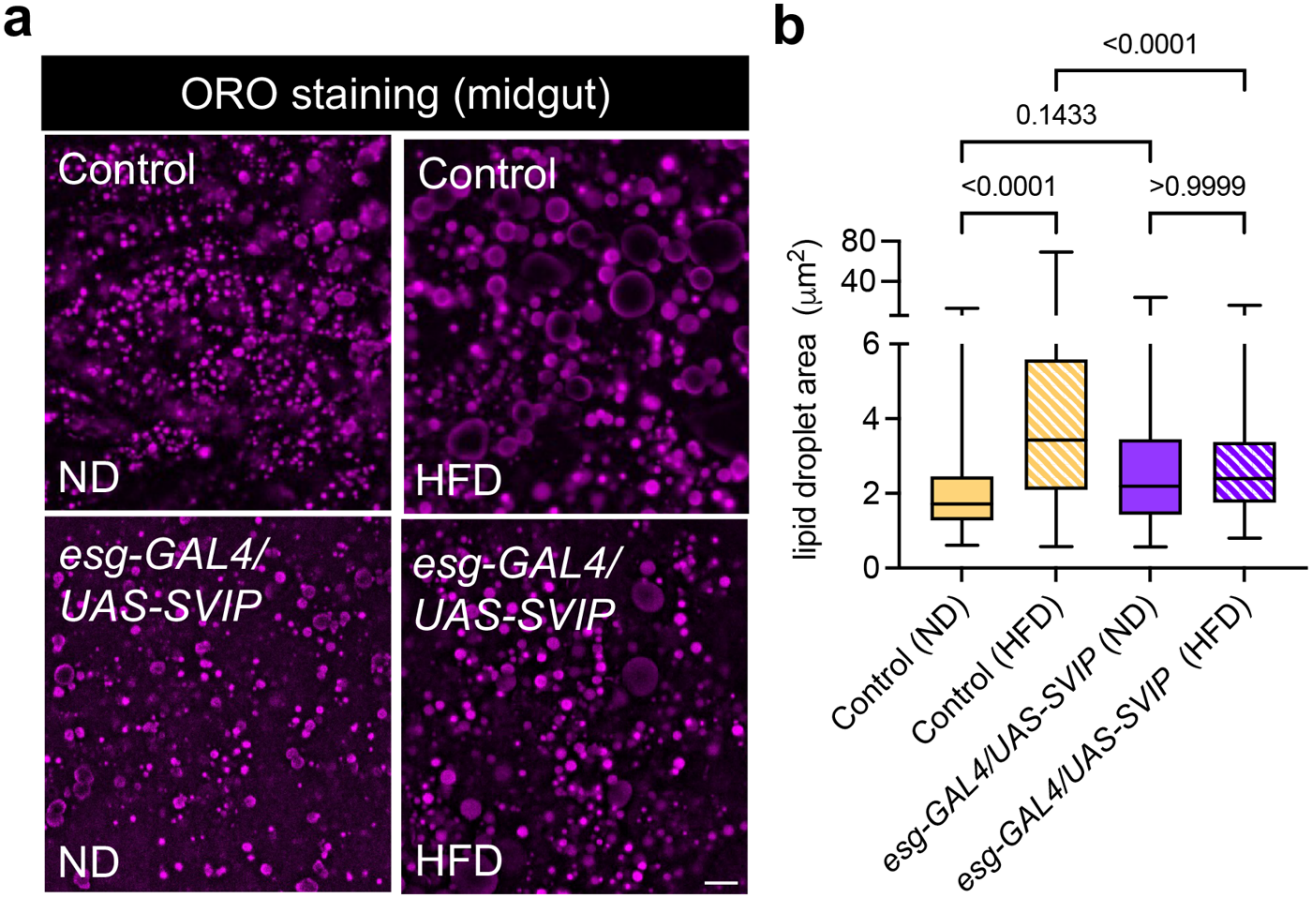
***SVIP* overexpression in the gut suppresses HFD-induced lipid droplet size increase.** (A) Representative images of Oil Red O (ORO) staining in the midgut of control flies and flies overexpressing *SVIP* in the gut that were fed a normal diet (ND) or high fat diet (HFD). Scale bar: 5 µm. (B) Quantification of lipid droplet area in the midgut of flies for the conditions and genotypes indicated (*n*=200 lipid droplets from 10 independent animals for each group). Data are presented as box-and-whisker plots, with horizontal lines inside boxes indicating medians, box edges representing 25th and 75th percentiles, and whiskers extending to minima and maxima. Statistical significance was determined using a two-way ANOVA with Šídák's multiple comparisons (*P*-values on graph).

Given that we observed an increase in lysosomal area (Fig. 1A,B), we surmised that lysosomes in the gut may become saturated under extreme HFD conditions, which could prevent lysosomes from optimally clearing cellular lipids at the primary site of digestion (gut). To assess this, we first examined lipid content in the gut of control flies that were fed either a normal diet (ND) or HFD. After 2 days on either diet, gut tissues were stained with Oil Red O (ORO) stain and the average area of lipid droplets were measured. As expected, we observed a significant increase in lipid droplet size in the gut of flies fed a HFD (Fig. 2A,B).

We next examined whether stimulating lysosome activity might be able to increase lipid turnover and prevent lipid accumulation in the gut. In previous work, we found that overproduction of the lysosomal adapter protein SVIP in both *Drosophila* and *C. elegans* can stimulate lysosome activity above basal levels by triggering a morphological shift to a tubular lysosomal network (Johnson et al., 2021; Villalobos et al., 2023). Therefore, we examined the effect of *SVIP* overexpression on lipid droplet size under HFD conditions. *SVIP* was overexpressed in the gut of flies using the *esg-GAL4* driver and flies were fed either a normal diet (ND) or HFD. Remarkably, in flies with *SVIP* overexpression in the gut, we observed no significant increase in lipid droplet size in flies that were fed a HFD for two days compared to flies fed a ND (Fig. 2A,B). These data suggest that *SVIP* overexpression can increase lipid turnover and prevent fat accumulation in the gut.

### *SVIP* overexpression in the gut improves lysosomal network integrity and increases lysosomal density in flies fed a HFD

**Fig. 3.**
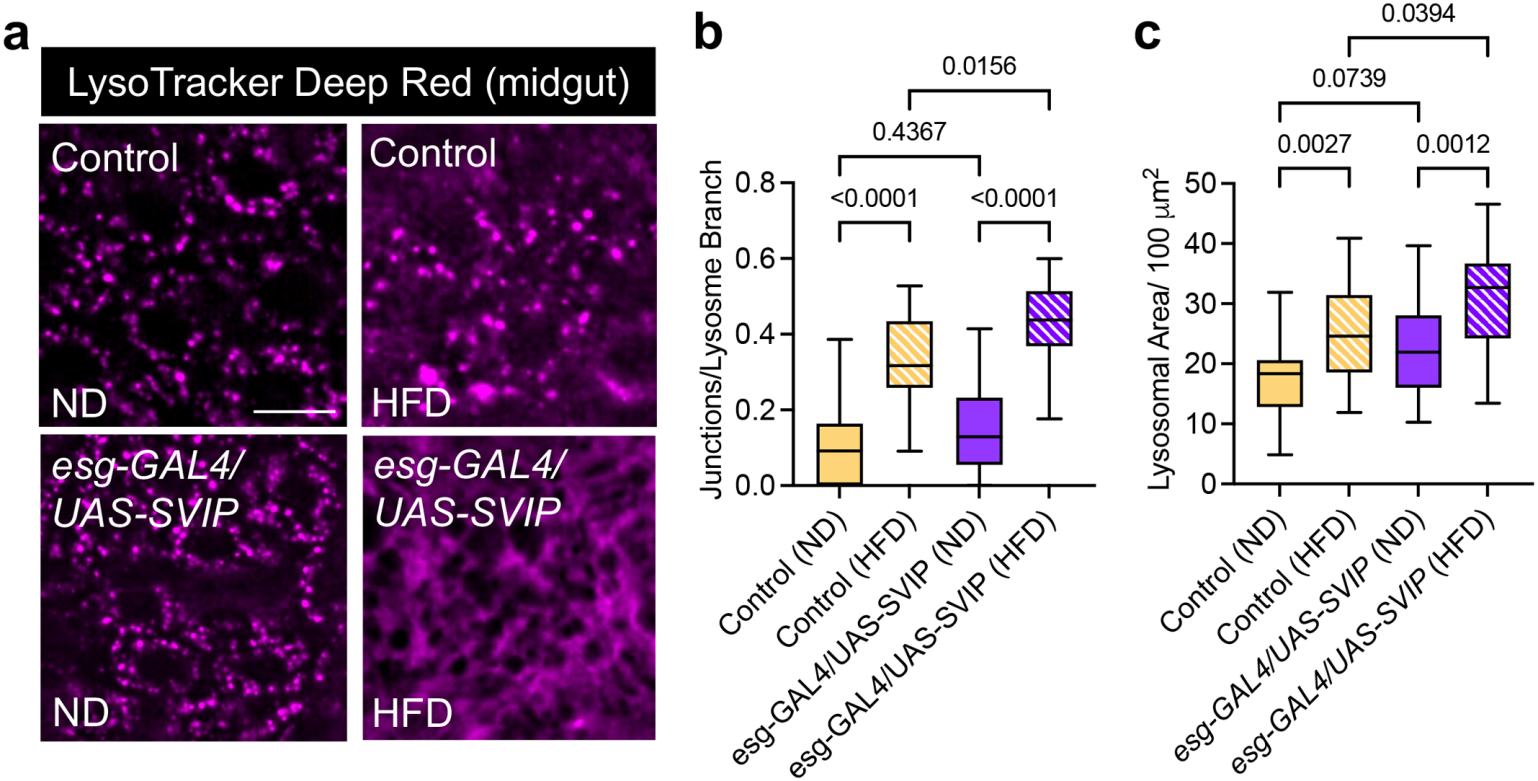
***SVIP* overexpression in the gut improves lysosomal network integrity and increases lysosomal density in flies fed a HFD.** (A) Representative images of LysoTracker Deep Red staining of the midgut of control flies or flies overexpressing *SVIP* in the gut that were fed a normal (ND) or high fat diet (HFD). Scale bar: 5 µm. (B) Quantification of lysosomal network junctions per branch for the indicated genotypes and conditions. (*n*=22 independent midguts for each group). (C) Quantification of lysosomal area per 100 µm^2^. (*n*=22 independent midguts for each group). Data are presented as box-and whiskers plots, with horizontal lines inside boxes indicating medians, boxes indicating medians, box edges representing 25th and 75th percentiles, and whiskers extending to minima and maxima. Statistical significance was determined using a two-way ANOVA with Sidak's multiple comparison (*P*-values on graph).

We next examined more directly how lysosomes respond to *SVIP* overexpression in flies fed a HFD. Flies were fed a ND or HFD for 2 days before gut tissues were dissected and imaged live. To visualize lysosomes, gut tissues were stained with the acidophilic dye LysoTracker. Consistent with our hypothesis, we found that *SVIP* overexpression in the gut of flies fed a HFD triggered a significant morphological shift in lysosomes. Specifically, we observed an increase in tubular networks and an increase in lysosomal density in flies fed a HFD with *SVIP* overexpression (Fig. 3A-C). Notably, we also observed an increase in tubular lysosomal networks in control flies fed a HFD alone, perhaps due to the increased lipids that need to be digested (Fig. 3A,B). In accord with this notion, we found in previous work that proteostatic stress is sufficient to trigger tubular lysosome induction (Villalobos et al., 2023). However, *SVIP* overexpression in combination with a HFD induced more robust tubular lysosomal networks compared to control flies fed a HFD and this correlates with significantly reduced lipid droplet size observed in flies fed a HFD with *SVIP* overexpression (Fig. 2A,B). Taken together, our data suggests that *SVIP* overexpression triggers robust tubular lysosomal networks that can accommodate the increased autophagic cargo load induced by a HFD.

### *SVIP* overexpression in the gut suppresses HFD-induced body mass increase

**Fig. 4.**
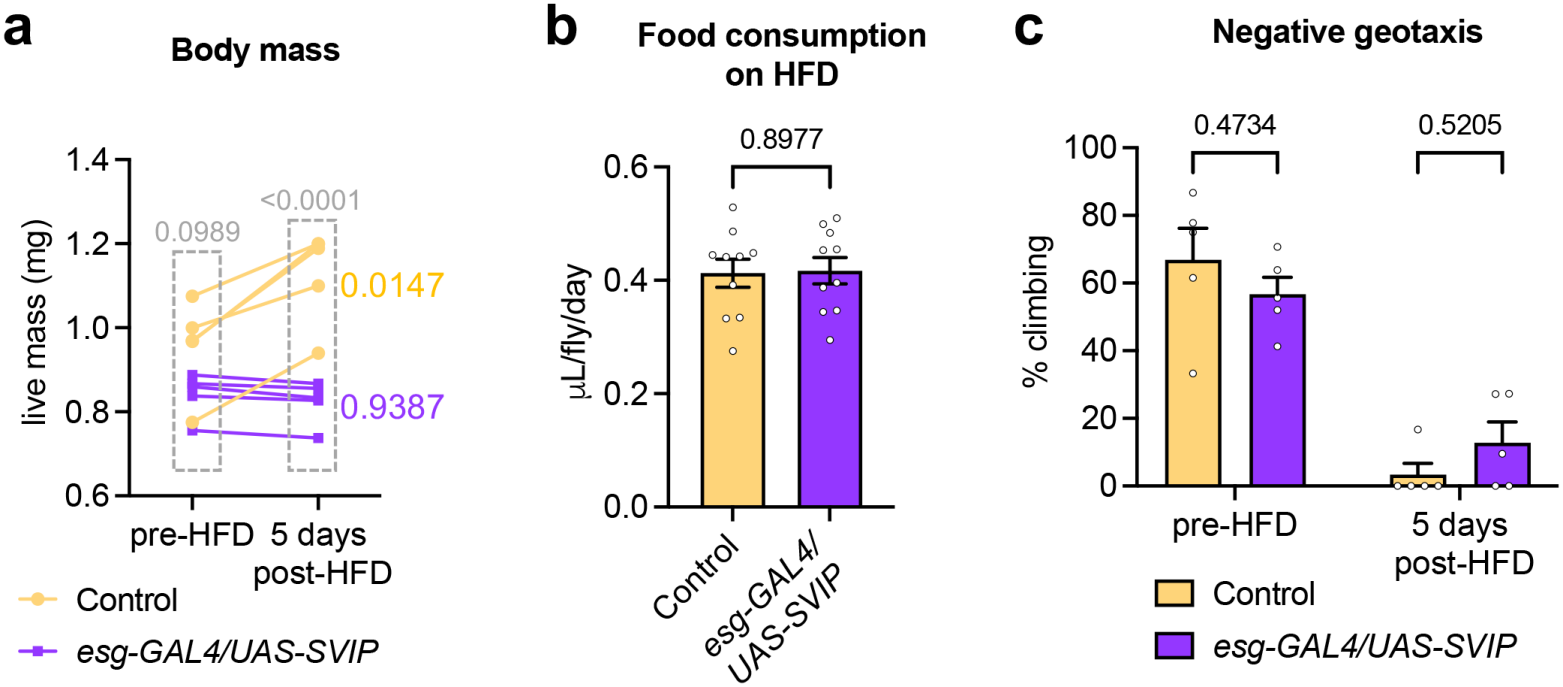
***SVIP* overexpression in the gut suppresses HFD-induced body mass increase.** (A) Average live weight of flies on a HFD overexpressing *SVIP* in the gut with the *esg*-GAL4 driver *(n*=4 replicates with 15 flies per replicate)*.* For each replicate, the same groups of flies were weighed in both the pre-HFD and 5 days post-HFD conditions. Data are presented as mean±s.e.m. Statistical significance was determined using two-way ANOVA with Šídák's multiple comparisons (*P*-values on graph). (B) Total food consumption per day of flies fed a HFD. (*n*=10 replicates with 20 flies per replicate). Data are presented as mean±s.e.m. Statistical significance was determined using an unpaired *t*-test (*P*-value on graph). c. Negative geotaxis assay of flies on a HFD overexpressing *SVIP* in the gut with the *esg*-GAL4 driver *(n*=5 replicates with 20 flies per replicate)*.* For each replicate, the same groups of flies were tested in both the pre-HFD and 5 days post-HFD conditions. Data are presented as mean±s.e.m. Statistical significance was determined using two-way ANOVA with Šídák's multiple comparisons (*P*-values on graph).

Because we observed suppression of fat accumulation in the gut, we next examined whether there were any organismal benefits to increasing lysosome activity in the gut via *SVIP* overexpression. In previous studies, a HFD has been shown to induce body mass increase in flies (de Paula et al., 2016). Thus, we measured changes in the live mass of control flies and flies overexpressing *SVIP* in the gut that were fed a HFD. The live mass of flies was measured prior to feeding a HFD (pre-HFD) and then again after 5 days on a HFD (5 days post-HFD). Because body mass is likely to vary between individual flies, the same groups of flies were measured at day one and then again at day 5 to directly assess changes in body mass of the same flies. As has been reported previously, control flies showed a significant increase in live body mass after 5 days on a HFD (Fig. 4A). Remarkably, *SVIP* overexpression in the gut completely suppressed the body mass increase in flies fed a HFD (Fig. 4A). Importantly, we verified that the reduction in body mass was not due to reduced food consumption; there was no significant difference in total food consumption per day in control flies and flies overexpressing *SVIP* in the gut (Fig. 4B).

We further examined the effect of increasing gut lysosome activity on other physiological parameters. Feeding flies a HFD generally leads to decreased locomotor activity (Baenas and Wagner, 2022; Liao et al., 2021), which can be assessed by conducting a negative geotaxis assay. Indeed, control flies that were fed a HFD demonstrated significantly reduced geotaxis after five days of consuming a HFD (Fig. 4C). Overexpression of *SVIP* did not significantly improve the mobility of flies fed a HFD (Fig. 4C). Thus, while stimulating lysosome activity in the gut can reduce overall body mass, it has no effect on improving locomotion.

Collectively, these data suggest that stimulating lysosome activity, specifically in the gut, can suppress the body mass increase observed in flies fed a HFD. Although the mechanism is unclear, we have demonstrated that *SVIP* overexpression suppresses lipid accumulation in the midgut (Fig. 2A,B), which could ultimately lead to less fat deposition and storage in other parts of the body.

### *SVIP* overexpression in the gut extends lifespan of flies fed a HFD

**Fig. 5.**
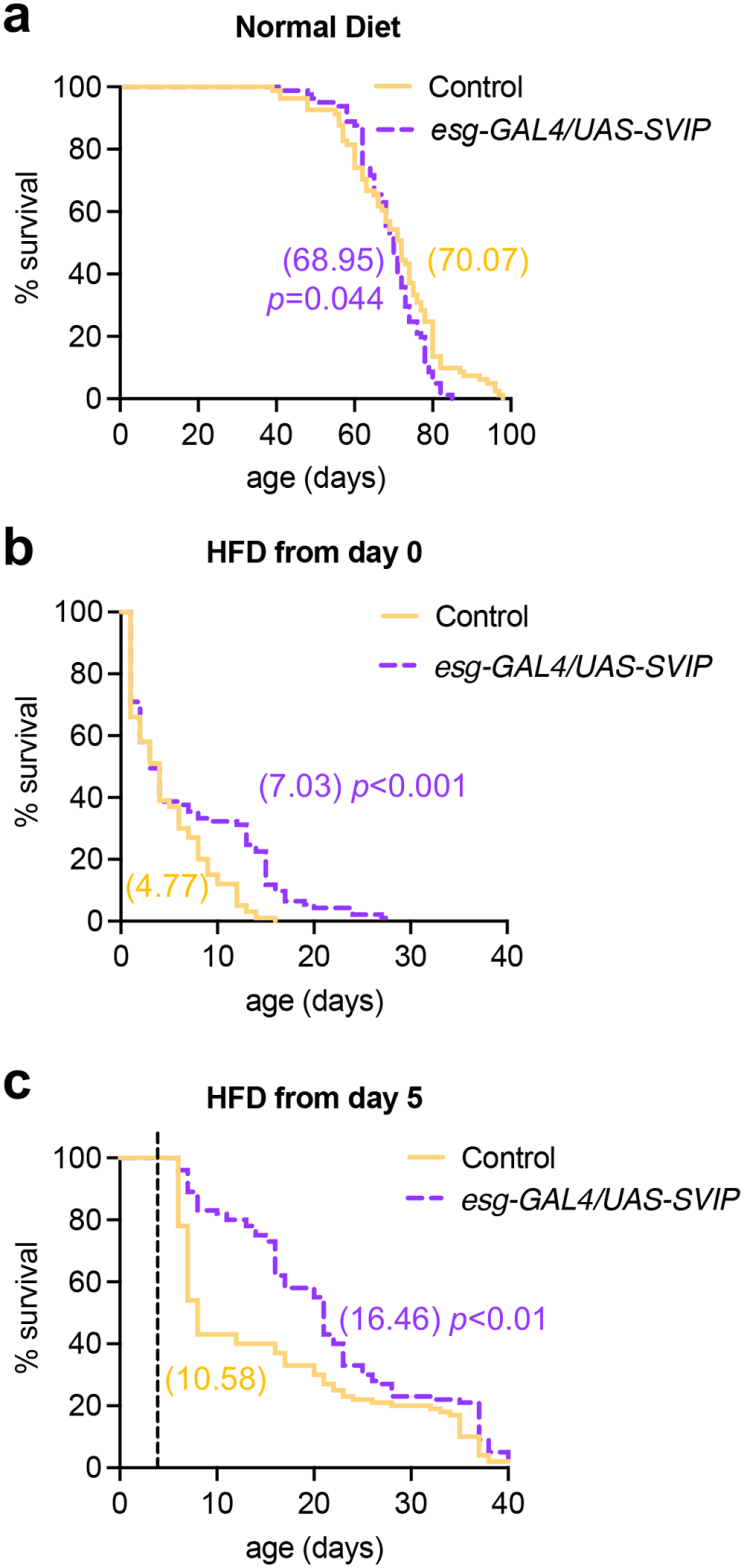
**SVIP overexpression in the gut extends lifespan of flies fed a HFD.** (A) Lifespan of control flies (*n*=100) and flies overexpressing *SVIP* in the gut with the *esg*-GAL4 driver *(n*=93) that were fed a HFD beginning at day 0 (first day of eclosion)*.* A log-rank test was used to determine statistical significance (*P*-values and mean lifespans are indicated on the plots). (B) Lifespan of control flies (*n*=100) and flies overexpressing *SVIP* in the gut with the *esg*-GAL4 driver *(n*=99) that were fed a HFD beginning 5 days post eclosion*.* A log-rank test was used to determine statistical significance (*P*-values and mean lifespans are indicated on the plots).

Finally, because we observed that *SVIP* overexpression could suppress body mass increase in flies fed a HFD, we examined whether *SVIP* overexpression in the gut could improve survival on a HFD. Newly eclosed flies were allowed to mate for 2-3 days on normal diet food to reach sexual maturity. After mating, male flies were isolated and transferred to either ND or HFD food. On a ND, *SVIP* overexpression did not significantly increase lifespan (Fig. 5A). This is consistent with our previous work demonstrating that overexpressing *Drosophila SVIP* in the gut of *C. elegans* does not significantly alter lifespan, but instead results in healthier aging (Villalobos et al., 2023).

We next examined whether *SVIP* overexpression in the gut could improve survivability of flies on a HFD. Although we observed an initial steep decline in survivability in both control flies and flies overexpressing *SVIP* in the gut, *SVIP* overexpression flies demonstrated improved survivability after five days on a HFD (Fig. 5B). This suggests that a HFD in the early part of adult *Drosophila* life might be more detrimental than when administered later. Potentially, there could be an early adult developmental event that is more sensitive to HFD conditions. To address this possibility, we assessed the effect on lifespan when flies were subjected to a HFD at a later point in adulthood. Newly eclosed flies were allowed to mate for 2-3 days on a normal diet. Subsequently, male flies were transferred to normal food for a period of 5 days before transferring them to HFD conditions. Notably, we observed a significant increase in initial survivability in flies overexpressing *SVIP* in the gut compared to control flies (Fig. 5C). Collectively, these results indicate that *SVIP* overexpression in the gut can improve survival of flies fed a HFD.

## DISCUSSION

Although obesity is often associated with a host of metabolic disorders and diseases, some individuals exhibit a remarkable resiliency that allows them to cope with certain metabolic stresses better than others. Given the increasing global prevalence of obesity, the search for genetic factors that can improve obesity tolerance is of considerable interest. Here, we have presented evidence that increasing lysosome activity, specifically in the gut via *SVIP* overexpression, is sufficient to suppress body mass increase and improve survival in flies fed a high-fat diet. Potentially, *SVIP* overexpression in the gut could be inducing more efficient turnover of lipids at the primary site of digestion and ultimately preventing fat deposition to other parts of the body. Moreover, increasing lysosome activity in the gut might allow organisms to better cope with extreme diets by promoting metabolic resiliency in the gut as well as other tissues.

Although future mechanistic studies will be important to clarify how *SVIP* overexpression and lysosome activity may be leading to these beneficial outcomes, there is increasing evidence that triggering autophagy–lysosome degradation can improve health of animals consuming high-fat diets. For example, dietary fasting practices (e.g. caloric restriction and intermittent fasting), which are robust autophagy stimulants, can reverse adverse metabolic states caused by a high-fat diet (Salgado-Canales et al., 2023; Barnosky et al., 2014). Moreover, alternating a protein-restricted diet with a high-fat diet improved healthy lifespan of male flies (Wang et al., 2024). Limiting protein in the diet of *Drosophila* inhibits mTOR signaling (Kapahi et al., 2004), which subsequently promotes autophagy-mediated degradation (Bouhamdani et al., 2021); thus, these results allude to the idea that activating autophagy can suppress the negative effects of a high-fat diet. Adding to this growing body of evidence, our results demonstrate that triggering lysosome activation also confers benefits to animals consuming high-fat diets. In previous work, we found that overexpression of *Drosophila SVIP* in the *C. elegans* gut triggers robust lysosome tubulation, which increases the rate of autophagic turnover by expanding the surface area and accessibility of the lysosomal compartment (Villalobos et al., 2023). By corollary, we propose that *SVIP* overexpression in the *Drosophila* gut suppresses obesity-related phenotypes by increasing autophagy proficiency.

Our results also highlight a specific gene, *SVIP,* that could be targeted for anti-obesity therapeutic approaches. *SVIP* encodes a small microprotein consisting of 82 amino acids in *Drosophila* and 77 amino acids in humans. Given the small size, it is conceivable that SVIP could be synthesized and delivered into cells exogenously. Notably, several protein domains with cell membrane penetrating properties have been discovered recently that can deliver peptides and even small proteins into cells via receptor-independent mechanisms (Heitz et al., 2009). For example, attachment of the HIV-TAT motif (YGRKKRRQRRR) was successful in delivering a 55 amino acid peptide (TONDU) into *Drosophila* tissues via oral administration (Bajpai et al., 2020). Similarly, *Drosophila* antennapedia homeoprotein (RKKRRQRRR) (Derossi et al., 1994) and the herpes simplex virus structural protein VP22 (Elliott and O'Hare, 1997) motifs have also been successful in delivering proteins as large as 27 kDa through eukaryotic cell membranes. Thus, an exciting possibility is that SVIP could be synthesized and administered as a dietary supplement.

Finally, although there are many limitations to our study, our results lead to some intriguing questions for future studies. Namely, would the combination of reduced protein diets in combination with *SVIP* overexpression produce an additive effect in suppressing obesity-related phenotypes? In previous work, we found that blocking lysosome tubulation in food-limiting conditions restricts their digestive capacity and prevents autophagy from reaching its full digestive potential (Villalobos et al., 2023). Thus, we would predict that stimulating earlier steps of autophagy via a protein-limited diet (or mTOR inhibition) in combination with lysosome tubulation would result in greater digestive capacity, which could be particularly beneficial in extreme dietary conditions. Another limitation of our study is that we did not explore the full repertoire of organismal health phenotypes that have been previously associated with obesogenic diets. For example, what effect does *SVIP* overexpression have on reproductive or transgenerational health? Obesogenic diets have a strong influence on germline health (Liu et al., 2022; Murashov et al., 2021), which can also lead to transgenerational effects in flies (Dew-Budd et al., 2016). Indeed, we found in earlier work that starvation triggers lysosome tubulation transgenerationally (Villalobos et al., 2023); thus, it is concievable that triggering lysosome tubulation could suppress obesity-related phenotypes in subsequent generations. Finally, our studies were conducted in male flies only, but it would also be interesting to test whether similar effects are observed in females given that many obesity-related traits exhibit strong sexual dimorphism (Murashov et al., 2021; Dew-Budd et al., 2016) and high-fat diets trigger different gene expression programs in male and female flies (Stobdan et al., 2019). Despite these experimental limitations, our results indicate that increasing lysosome digestive capacity can offset some of the negative impacts of a high-fat diet and highlight a potential genetic target for boosting metabolic resiliency under extreme dietary conditions.

## MATERIALS AND METHODS

### *Drosophila* husbandry and diets

Flies were maintained at 25°C in a 12 h:12 h light: dark cycle for all experiments. Normal diets contained 6% (w/v) cornmeal (VWR, 75860-346), 1.5% (w/v) yeast (Genesee Scientific, 62-107), 1% (w/v) agar (Genesee Scientific, 66-105), 8% (v/v) molasses (VWR, 75860-374), 0.8%(v/v) Tegosept (Thermo Fisher Scientific, NC0238407), 0.24% (v/v) propionic acid (Thermo Fisher Scientific, BPA258500), and 0.02% (v/v) phosphoric acid (Sigma-Aldrich, PX09956). For high fat diets, Organic Coconut Oil (Glorybee) was added to normal diet food at 15% (w/v). For high-sugar diets, D-Sucrose (Thermo Fisher Scientific, BP220-1) was added to the normal diet food at 30% (w/v). Fly stocks include *esg*-GAL4, mCD8-GFP (lab stock), *UAS-spin-myc-RFP* (BDSC, 39668), *UAS-SVIP* (Johnson et al., 2021).

### Lysosome imaging

The midgut were dissected from live male flies in O'Dowd's saline buffer [101 mM NaCl, 4 mM MgCl_2_, 3 mM KCl, 5 mM glucose, 1.25 mM NaH2PO4, and 20.7 mM NaHCO3, pH 7.2] (Gu and O'Dowd, 2006). Tissues were immediately mounted on a Gold Seal™ glass microscope slide (Thermo Fisher Scientific) with saline and a glass cover slip (Thermo Fisher Scientific). Imaging was performed with a Leica DMi8 wide-field fluorescence microscope equipped with 10X (NA 0.32), 40X (NA 1.30), and 100X (NA 1.40) objectives. Spin-RFP was imaged using a Texas Red filter set. For LysoTracker staining, tissues were incubated at room temperature in LysoTracker™ Deep Red (ThermoFisher, L12492) diluted 1:1000 in saline solution for 30 min. The tissues were then washed once for no more than 60 s in saline. They were then mounted on a glass microscope slide (Thermo Fisher Scientific) in saline, covered with a glass cover slip (Thermo Fisher Scientific) and sealed with Vaseline. Imaging was performed with a Leica DMi8 wide-field fluorescence microscope using a 100× (NA 1.40) objective, a far-red filter, and THUNDER imager.

### Oil Red O staining

A 5 mg/ml working stock solution of Oil Red O (Alfa Aesar, A12989) was filtered using a 0.45 μm syringe filter (Fisherbrand, 09-720-514) to remove crystals. The working solutions were diluted at a 3:2 ratio with DI water. Gut tissues were dissected from live animals in 1× phosphate-buffered saline (PBS; 137 mM NaCl, 2.7 mM KCl, 10 mM Na_2_HPO_4_, 1.8 mM KH_2_PO_4_) and then washed in PBS-T [1× PBS, 0.1% Tween-20]. Midgut tissues were dissected from male flies and fixed in 100 µl of 60% Isopropanol for 3 min with gentle rocking. Subsequently, 600 µl of ORO working stock solution was added and the tube was inverted 3 times. Samples were incubated for 2 h on a nutator in the dark and then washed with PBS-T before imaging.

### Image analysis

Lipid size and lysosome size were measured 20 times at random in a region of interest (ROI) from 10 independent midgut tissues. ImageJ/FIJI was used to measure the area of lysosomes and lipid sizes. For tubular lysosome network analysis, a 100 µm^2^ ROI was analyzed for each image. For tubular lysosomal network integrity, networks were analyzed using the ‘skeleton’ analysis plug-ins in FIJI (National Institutes of Health). In short, each image was converted to 8-bit binary images and then were converted to skeleton images using the ‘Skeletonize’ plugin. Finally, the skeleton images were quantified using the ‘analyze skeleton’ plug-in. The number of lysosomal junctions per branch was scored. The lysosomal network density was quantified using the same ROI as the TL integrity analysis. Images were converted to 8-bit binary images and the area or volume of the image occupied by the signal intensity was calculated using the ‘Create Selection’ plugin that calculates the area of the fluorescent signal.

### Live mass measurements

For each biological replicate, 15 male flies were transferred to a 1.5 ml Eppendorf tube and the live weight of all 15 flies was measured. The total weight was divided by 15 to calculate the average live mass per fly per replicate.

### Food consumption assay

Food consumption experiments were adapted from a protocol that has been previously described (Shell et al., 2018). Briefly, Blue #1 dye (FD and C Blue #1, Spectrum Chemical, FD110-25GM) was dissolved into HFD agar food at a concentration of 1% (w/v). 4 ml of food was dispensed into feeder caps (MOCAP, FCS.813NA1). Three to 5-day-old male flies were transferred into empty K-resin narrow fly vials (15 flies/vial) and the feeder caps were placed on top of the vials. Vials were placed on their side since the HFD food makes the sides of the vials greasy and prevents flies from climbing to the top of the vials. Flies were allowed to consume food for 24 h. After 24 h, flies in each vial were collected and homogenized into 0.5 ml of water, the debris was pelleted, and the supernatant was transferred to a fresh tube. 1 ml of water was added to each tube and vortexed to mix (internal dye, INT). The dye excreted in the vials was collected by washing the tubes with 3 ml water (excreted dye, ExVial). Note, any vials with dead flies were censored. The absorbance at 630 nm for the INT and exVial was measured for each replicate using a UV–VIS spectrophotometer. Absorbances were converted to volumes by generating a standard curve using pure dye. Total consumption was measured by combining INT+ExVial.

### Negative geotaxis assay

For each biological replicate, 20 male flies were transferred to a fresh vial with no food. The number of flies that could climb 3 cm in 10 s were scored. For each replicate, the same groups of flies were tested in the pre-HFD and post-HFD conditions.

### Lifespan analysis

Newly eclosed flies were allowed to mate on a normal diet for 2-3 days. After 2-3 days, male flies were isolated and transferred to vials (20 flies/vial) on either normal diet or high fat diet food. Flies were transferred to fresh vials every 1-2 days and deaths were scored at the time of transfer. Lifespan data was analyzed with OASIS 2 software (Han et al., 2016) and a log-rank test was used to determine statistical significance.

### Statistical analyses

All statistical analyses were performed with GraphPad Prism 8. Two-way ANOVAs and unpaired *t*-tests were performed under a 5% significance level.
